# Low potential for mechanical transmission of Ebola virus *via* house flies (*Musca domestica*)

**DOI:** 10.1186/s13071-017-2149-x

**Published:** 2017-05-03

**Authors:** Andrew D. Haddow, Farooq Nasar, Christopher W. Schellhase, Roger D. Moon, Susana L. Padilla, Xiankun Zeng, Suzanne E. Wollen-Roberts, Joshua D. Shamblin, Elizabeth C. Grimes, Justine M. Zelko, Kenneth J. Linthicum, Sina Bavari, M. Louise Pitt, John C. Trefry

**Affiliations:** 1 United States Army Medical Research Institute of Infectious Diseases, 1425 Porter Street, Frederick, MD 21702 USA; 2 Department of Entomology, University of Minnesota, 219 Hodson Hall, 1980 Folwell Avenue, St. Paul, MN 55108 USA; 3 United States Department of Agriculture, Agricultural Research Service, Center for Medical, Agricultural, & Veterinary Entomology, 1600 SW 23rd Drive, Gainesville, FL 32608 USA

**Keywords:** Ebola virus, Filovirus, *Musca domestica*, Fly, Muscid, Mechanical, Fomite, Transmission, Outbreak

## Abstract

**Background:**

Ebola virus (EBOV) infection results in high morbidity and mortality and is primarily transmitted in communities by contact with infectious bodily fluids. While clinical and experimental evidence indicates that EBOV is transmitted *via* mucosal exposure, the ability of non-biting muscid flies to mechanically transmit EBOV following exposure to the face had not been assessed.

**Results:**

To investigate this transmission route, house flies (*Musca domestica* Linnaeus) were used to deliver an EBOV/blood mixture to the ocular/nasal/oral facial mucosa of four cynomolgus macaques (*Macaca fascicularis* Raffles). Following exposure, macaques were monitored for evidence of infection through the conclusion of the study, days 57 and 58. We found no evidence of systemic infection in any of the exposed macaques.

**Conclusions:**

The results of this study indicate that there is a low potential for the mechanical transmission of EBOV *via* house flies - the conditions in this study were not sufficient to initiate infection.

## Background

In December 2013, Ebola virus (EBOV) emerged in West Africa initiating an epidemic that resulted in more than 28,600 cases and 11,300 deaths [1]. While the primary mechanism of EBOV transmission is through human contact with infectious bodily fluids originating from infected patients or cadavers [2, 3], other potential mechanisms of transmission warrant investigation. During the height of the outbreak questions were raised in regards to the ability of non-biting muscid flies to mechanically transmit EBOV due to the high number of cases, regional mourning and funeral practices, unsanitary conditions and a lack of vector control.

The mechanical transmission of viruses involves the transfer of virions by a vector to a person or animal through direct contact in the absence of multiplicative replication, and can occur following direct contact with virus-contaminated mouthparts, legs and/or the body of an arthropod. The mouthparts of non-biting muscid flies are not designed to pierce or damage the skin to permit blood-feeding, rather they are designed to lap-up fluids [4]. However, non-biting muscid flies will feed on blood if it is accessible, in the case of wounds [4]. Furthermore, they will not only feed on body secretions, but may preferentially seek them out [5]. For example, the principle vector of trachoma (granular conjunctivitis) in children, *Musca sorbens* Wiedemann, mechanically transmits the bacterium by feeding on mucus and discharge from infected eyes and then transferring it to an uninfected host [5]. Such behaviors among non-biting muscid flies have been shown to be responsible for the mechanical transmission of numerous other bacterial and viral pathogens [5–7].

To determine the ability of non-biting muscid flies to mechanically transmit EBOV, we allowed house flies (*Musca domestica* Linnaeus) to walk in moderate- to high-dose EBOV/blood mixtures and then walk on the faces (area around the eyes, the nose and the lips) of cynomolgus macaques (*Macaca fascicularis* Raffles).

## Methods

Research was conducted under an IACUC-approved animal protocol at the United States Army Medical Research Institute of Infectious Diseases (USAMRIID). This protocol complied with the Animal Welfare Act, Public Health Service Policy, and other Federal statutes and regulations relating to animals and experiments involving animals. The facility where this research was conducted is accredited by the Association for Assessment and Accreditation of Laboratory Animal Care, International and adheres to principles stated in the Guide for the Care and Use of Laboratory Animals, National Research Council, 2011.

*Musca domestica* were descendants (F30-40) of adult flies netted at the University of Minnesota dairy barn at the St. Paul campus in July 2014, and were reared with methods adapted from Moon et al. [8]. Adults for all experiments were reared from puparia shipped overnight to USAMRIID. Upon arrival at USAMRIID pupae were placed into gallon sized cardboard cartons with netting and kept at 28°C, approximately 80% RH and a photoperiod of 12:12 h light/dark. Upon emergence, adult flies were provided sugar cubes and water. Due to logistic complexities male and female flies were not separated and all experiments utilized a combination of male and females.

Just prior to the experiment, adult *house flies* were briefly chilled at -20°C to immobilize them and were then placed on an ice-chilled Petri dish. Flies had their wings cut-off to prevent flight and a string was attached to the dorsal side of the thorax using cyanoacrylate superglue. Individual flies were placed into 50 ml conical tubes containing a piece of seed germination paper to allow the flies a location to rest. The top of each conical tube was covered with mesh (extended beyond the opening to permit easy removal) and secured with a rubber band. Half of the string was allowed to remain on the outside of the tube. The string was long enough to be easily gripped with one gloved hand, while still being able to remove the rubber band and mesh with the other gloved hand. Flies in tubes were provided a 10% sucrose solution *via* a cotton ball until they were experimentally utilized.

Since, to our knowledge, this was the first study to investigate mechanical transmission of a viral pathogen by dipterans in a BSL-4 laboratory, a risk assessment was performed by the experimental team and biosafety personnel prior to the study. The use of a downdraft table or biosafety cabinet was ruled out due to airflow conditions that would disrupt the ability of the fly to walk along facial surfaces. Additionally, there were concerns that the airflow would lead to the rapid desiccation of the flies themselves. Limited space precluded the use of a dedicated glove box. It was therefore decided to perform the experiment on a procedure table. During the experiment one person solely observed and monitored fly-walking and was prepared to catch and kill any flies that detached from a tether, while the other two personnel walked flies.

**Fig. 1:**
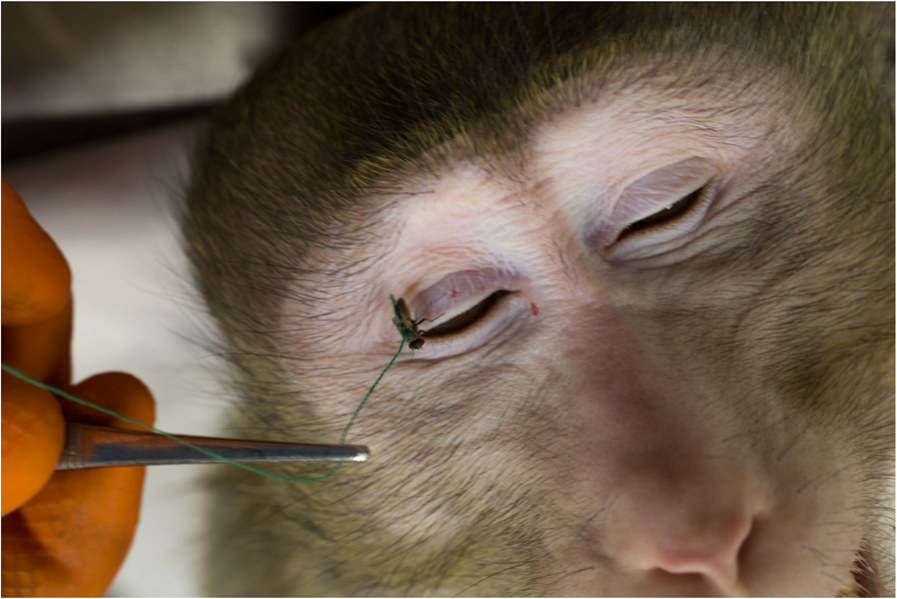
A house fly exposed to an Ebola virus/whole blood mixture walking on the eyelids of a cynomolgus macaque. Visible blood spots are present on both the right upper and lower eyelid

We exposed four adult cynomolgus macaques (negative for filovirus antibodies), three males (macaques 1, 3 and 4) and one female (macaque 2) to tethered house flies (Fig. 1) that had been exposed to a thin layer of EBOV (Kikwit strain, 7U variant, GenBank accession number KT762962) mixed with whole blood drawn from each individual macaque (1:1 ratio). The virus/blood mixture used for each experiment, as well as a sub-set of homogenized whole flies (3 per macaque) exposed to the virus/blood mixture, were titered on Vero E6 cells by plaque assay [9] (Table 1). Individual tethered flies were then allowed to rest, walk and/or imbibe the virus/blood mixture for a period of 15 s; they were then transferred to an individual macaque and allowed to rest, walk and/or imbibe secretions for a period of 30 s (Fig. 1). The target number of flies for each macaque was 40 (10 each eye, 10 nose, 10 mouth), but fly mortality reduced the total number of flies for macaques 1 (10 each eye, 7 nose, 9 mouth) and 2 (10 each eye, 9 nose, 7 mouth). The area around the eye included the upper and lower eyelid, the lacrimal papilla, the plica semilunaris and the upper and lower eye lashes. The area of the nose included the supratip break to the philtrum, ending at the ala of the nose. The area of the mouth included the upper and lower lip, the corners of the mouth, the cutaneous edge of the mouth and the lips.

**Table 1:** Titers of Ebola virus/whole blood mixtures and exposed house flies

Macaque	Virus/blood titer (PFU/ml)	Fly	Whole fly titer (PFU/fly)
1	7.8 × 10^6^	1	1
		2	0^a^
		3	95
2	6.8 × 10^6^	1	68
		2	1
		3	13
3	2.3 × 10^6^	1	10
		2	0^a^
		3	0^a^
4	5.0 × 10^5^	1	0^a^
		2	5
		3	0^a^

^a^Limit of detection of 1 PFU/fly

Post-exposure, macaques were evaluated daily for signs of illness [9]. Using standard techniques [9], blood chemistry, hematology, rectal temperature and body weight were performed and/or taken under anesthesia on days: pre-challenge, 0, 3, 6, 8, 10, 14, 21, 28, 35, 42, 49 and terminal (57/58). Free catch drool/saliva was collected daily under anesthesia from day 8 to 21 (except day 11) and on days 28, 35 and 42.

All macaques survived to the end of study and were euthanized following standard procedures on days 57 (macaques 3 and 4) and 58 (macaques 1 and 2) [9]. Following euthanasia, the submandibular lymph node, submandibular salivary gland, lungs, tongue, tonsil, trachea, esophagus, thyroid gland, tracheobronchial lymph node, heart, liver, spleen, kidneys, ileum, cecum, colon (proximal and distal), brain, eye, haired skin, nares/lip, maxilla and nasal cavity were collected from each macaque and fixed using standard techniques [9].

EBOV immunohistochemistry, IgG ELISA and RT-qPCR were performed using standard techniques described previously [9].

## Results

Flies were observed to walk and occasionally feed on mucosal secretions. Visible residual EBOV/blood was observed on the skin following exposure of some macaques (Fig. 1). Virus/blood titers used to expose flies ranged from 5.0 × 10^5^ to 7.8 × 10^6^ PFU/ml and whole fly titers ranged from at (1 PFU/fly) or below the limit of detection to 95 PFU/fly (Table 1). During the course of the experiments no flies detached from the tether. The airflow in the procedure room (i.e. lack of humidity) did result in a high mortality of flies awaiting experimental use.

None of the exposed macaques presented with any observable clinical signs of EBOV disease, and chemistry and hematology results were normal. EBOV antibodies and vRNA in the sera were not detected at any time point through day 42. vRNA was detected by qRT-PCR in saliva at low levels on day 21 (macaques 3 and 4) and 28 (macaque 3). The mean Ct values of triplicate runs were 34.2 (animal 3, day 21), 34.2 (animal 4, day 21) and 34.4 (animal 3, day 28), with a cut-off of 36.0. All negative extraction and negative PCR controls were negative.

No lesions suggestive of filovirus infection were observed at gross necropsy or by histologic examination. Tissues assayed by immunohistochemistry for filoviral antigen were negative.

## Discussion

In tropical environments, such as West Africa, non-biting muscid flies are ubiquitous and closely associated with human populations. Increased unsanitary conditions, such as those that occurred at the height of the West African EBOV outbreak, would have exacerbated fly populations increasing fly contact with EBOV infective persons, body fluids, wastes and/or cadavers. Therefore, we modeled the mechanical transmission potential of EBOV by house flies using immunologically naïve cynomolgus macaques.

In this study, we demonstrated there is considerable variation regarding the amount of EBOV/blood that an individual fly is exposed to under experimental conditions (0–95 PFU per fly). This variation is likely due to a combination of the external morphology and behavior of flies, as well as the viscosity and titer of infectious blood. The mouthparts, wings, abdomen, legs and pulvilli of flies are all capable of providing adhesive surfaces for pathogen transport following contact with contaminated fluids [10]. In our study, experimental flies were observed to engage in a variety of behaviors including walking, hopping, remaining motionless, visible feeding or lack thereof and/or the resting of the abdomen in the EBOV/blood mixture resulting in varying degrees of adhesion to anatomical structures. Additionally, the ingestion of EBOV/blood by some flies may have increased the detected titer. All of these factors would have contributed to the variation in virus titers observed from whole flies (Table 1). However, such variation would also occur in nature leading to the potential for no EBOV/blood adhesion to moderate/high adhesion depending on exposure time, viscosity of the infectious fluid and the virus titer of the infectious fluid.

Based on our observation of a maximum titer of 95 PFU/fly, we estimate the potential maximum experimental dose for a 40 fly EBOV/blood exposure event to be 3.8 × 10^3^ PFU/fly. The amount of virus deposited on a host would also vary considerably due to the factors noted above, as well as desiccation of the infectious fluid on the anatomical structures of a fly and the physical properties of animal skin. Our study attempted exposure *via* a one-time event where the faces of macaques (i.e. ocular, nasal and oral mucosa) were exposed to 36–40 EBOV/blood-exposed house flies. Although it is unlikely that every non-biting muscid fly present in the vicinity of an infectious EBOV patient would have been exposed to infectious bodily fluids, it is probable that patients would be exposed to continuous fly landings. Field studies have shown *M. sorbens* to have landing rates as high as 8.5 flies/min on faces [11] and this species has been shown to land of the faces of children with nasal or ocular discharge twice as often as children with clean faces [5]. Although our study did not demonstrate mechanical transmission of EBOV, repeated EBOV/fly exposures may reach the 100 PFU/ml threshold that has been shown to result in muscosal transmission [12]. Therefore, persons working in areas with high burdens of non-biting muscid flies and potentially infectious persons, body fluids, wastes and/or cadavers should wear appropriate PPE.

Neither of the macaques that had detectable vRNA in the free-catch saliva/drool at days 21 and 28 post-exposure had detectable vRNA in the blood at any time point, seroconverted or exhibited evidence of EBOV infection in any of the tissues assayed by IHC following necropsy, including immunologically privileged tissues. It is possible that the EBOV vRNA positive saliva/drool specimens may have come into contact with the initial areas of fly exposure (i.e. upper and lower lips, the corners of the mouth and the cutaneous edge of the mouth and lips) and that the RT-qPCR signal did not result from vRNA within the mouth. These findings are similar to those of Mire et al. [12] who detected EBOV vRNA in the nasal swab of a cynomolgus macaque at a single time point (day 10) in the absence of seroconversion following a 10 PFU/ml EBOV droplet (Makona strain) inoculation to oropharynx. As such, it appears mechanical transmission of EBOV *via* house flies is very inefficient, as seen in other viruses involving fomite transmission [13]. Our results are consistent with those of Bausch et al. [14], who determined that there was a low risk of EBOV transmission *via* fomites; as well as those of Mire et al. [12] who demonstrated that low-dose oral and conjunctival EBOV exposure did not result in clinical infection in cynomolgus macaques.

## Conclusions

In summary, we demonstrated that there is a low potential for the mechanical transmission of EBOV *via* house flies - the conditions in this study were not sufficient to initiate infection.

## Abbreviations

BSL-4: Biosafety level 4 laboratory
Ct value: Threshold cycle
EBOV: Ebola virus
ELISA: Enzyme-linked immunosorbent assay
IACUC: Institutional animal care and use committee
RH: Relative humidity
RT-qPCR: Real-time quantitative reverse transcription polymerase chain reaction
USAMRIID: United States Army Medical Research Institute of Infectious Diseases
vRNA: Viral ribonucleic acid
